# Contributions of Fasting and Postprandial Glucose Concentrations to Haemoglobin A1c in Drug-Naïve Mal-Glucose Metabolism in Chinese Population Using Continuous Glucose Monitoring System

**DOI:** 10.1155/2019/1267475

**Published:** 2019-12-01

**Authors:** Rengna Yan, Yun Hu, Fengfei Li, Lanlan Jiang, Xiaohua Xu, Jie Wang, Ying Zhang, Lei Ye, Kok Onn Lee, Xiaofei Su, Jianhua Ma

**Affiliations:** ^1^Department of Endocrinology, Nanjing First Hospital, Nanjing Medical University, Nanjing 210012, China; ^2^National Heart Research Institute Singapore, National Heart Centre Singapore, Singapore 169609; ^3^Department of Medicine, National University of Singapore, Singapore 119228

## Abstract

**Aim:**

To clarify the contributions of fasting glucose (FG) and postprandial glucose (PG) to HbA1c in drug-naïve patients with type 2 diabetes (T2D) and impaired glucose tolerate (IGT)/impaired fasting glucose (IFG).

**Methods:**

Continuous glucose monitoring (CGM) was performed in 305 drug-naïve Chinese patients with T2D or IGT/IFG. The incremental area under the curve (AUC) above a glucose value of 6.1 mmol/L or FG glucose levels were calculated to evaluate the contributions of PG or FG to HbA1c values.

**Results:**

According to quintiles of HbA1c, T2D patients were divided into five groups (group 1 to 5), and patients with IGT/IFG were assigned into group 0. PG was the predominant contributor in the lower groups with HbA1c 4.9∼6.0% and 6.1∼7.8%. The relative contributions of FG and PG to HbA1c had no significance in the middle groups of HbA1c (7.9∼8.7% and 8.8∼9.5%). FG contributed significantly more than PG in the higher groups of HbA1c (9.6∼10.9% and 11.0∼14.6%). Regression analyses indicate that the contributions of FG and PG were equal (both 50%) when the level of HbA1c was 8.5%.

**Conclusions:**

In drug-naïve patients with T2D or IGT/IFG, PG contributed more in patients with HbA1c < 8.5%, whereas FG became the predominant contributor in the poorly controlled patients with HbA1c ≥ 8.5%. These results may help the health-care provider set appropriate plasma glucose testing goals with the expectation of achieving specific HbA1c values.

## 1. Introduction

Glycemic control is a cornerstone in delaying the onset and decreasing the incidence of both the short- and long-term complications of diabetes. Glycated haemoglobin (HbA1c) is a gold-standard parameter in evaluating the effectiveness of glycaemic control in patients with diabetes [1]. The Diabetes Control and Complication Trial (DCCT) and the United Kingdom Prospective Diabetes Study (UKPDS) indicated that HbA1c > 7.0% is associated with a significantly increased risk of both microvascular and macrovascular complications, regardless of underlying treatment [2–5]. Fasting glucose (FG) and postprandial glucose (PG) are commonly used as daily makers to evaluate glycaemic control and modify therapeutic strategy. Clearly understanding the relationship between plasma glucose levels and HbA1c will help to set appropriate day-to-day plasma glucose testing goals for achieving the target HbA1c level. There is no consensus about the association between FG and PG with HbA1c in patients with type 2 diabetes (T2D) [6–14]. Most previous studies were based on multipoint glucose measurements in treated patients. In this study, we evaluate the contribution of FG and PG on HbA1c in newly diagnosed and untreated patients with T2D or IGT/IFG using continuous glucose monitoring systems (CGMS). This may help to identify the better surrogate glycemic marker for achieving the target HbA1c level and for early detection of glycemic control status.

## 2. Materials and Methods

### 2.1. Subjects

According to 1999 WHO diagnostic criteria [15], DM was defined as fasting plasma glucose ≥7.0 mmol/L and/or 2 h PG ≥ 11.1 mmol/L. Impaired Glucose Tolerance (IGT) was defined as fasting plasma glucose <7.0 mmol/L and 2 h PG ≥ 7.8 mmol/L. Impaired Fasting Glycaemia (IFG) was defined as fasting plasma glucose ≥6.1 to <7.0 and 2 h PG < 7.8 mmol/L. A total of 305 newly diagnosed, drug-naïve patients with T2D or IGT/IFG were recruited between January 2015 and December 2018 in Nanjing First Hospital, Nanjing Medical University, China. The inclusion criteria were newly diagnosed, drug-naive patients with T2D or IGT/IFG. Patients who had ketoacidosis, impaired renal (serum creatinine > 150 *μ*mol/L) or liver (aspartate aminotransferase or alanine aminotransferase 2.5 times the upper limit of the normal range) functions or a history of anaemia, or suffered from cancer were excluded. The study was approved by the ethics committee of Nanjing Hospital. Informed consents were signed by all patients. The methods were conducted in accordance with the Declaration of Helsinki guidelines, including any relevant details.

### 2.2. Clinical and Laboratory Assessments

After admission, detailed interviews and regular laboratory analyses were performed in all patients. Anthropometric parameters of height, weight, waist circumference, hip circumference, and blood pressure were measured, and recorded BMI was calculated as body weight divided by height squared (kg/m^2^). HbA1c was analyzed using high-performance liquid chromatography (Bio-Rad, USA). Serum creatinine, liver functional parameters (aspartate aminotransferase and alanine aminotransferase), and lipid profiles (total cholesterol, triglycerides, high- and low-density lipoproteins) were measured by enzymatic assays (Olympus AU5400 autoanalyzer; Beckman Coulter, Japan).

### 2.3. Food Intake

All subjects were instructed to maintain physical activity according to their doctors' personalized instructions and received meals consisting of a total daily caloric intake of 25 kcal/kg/day. The ratio of carbohydrate, proteins, and fats were 55%, 17%, and 28%, respectively. Patients were instructed to have breakfast, lunch, and dinner at 7 : 00 AM, 11 : 00 AM, and 5 : 00 PM, respectively.

### 2.4. Calculation of the Relative Contributions of PG and FG to A1c Levels

A retrospective CGMS (Sofsensor, CGMS-Gold, Medtronic Incorporated, Northridge, USA) [16] was applied to the recruited patients 72 hours before glycemic control treatment. To minimize the influence of diet taken before hospital admission, the contributions of FG and PG to overall hyperglycaemia was calculated using CGMS data obtained in the second 24 hours (24 h) after admission. Blood glucose level >6.1 mmol/L is considered as hyperglycemia. We defined the glucose area under the curve (AUC) above 6.1 mmol/L during the second 24-h interval as AUC_total_ to represent overall hyperglycaemia [13]. The glucose AUC above FG was defined as AUC_PG_ reflecting the contribution of PG to overall hyperglycaemia during 24 h. The AUC_FG_ was defined as AUC_total_ − AUC_PG_. The relative contributions of FG and PG to overall hyperglycaemia were calculated as ((AUC_total_ − AUC_PG_)/AUC_total_) × 100% and (AUC_PG_/AUC_total_) × 100%, respectively [14]. The HbA1c values used for analysis in this study were those obtained at admission. According to quintiles of HbA1c, the subjects with diabetes were divided into five groups (from 1 to 5) and patients with IGT/IFG were allocated into group 0 to evaluate the contribution of FG and PG to glucose increments.

### 2.5. Statistical Analysis

All statistical analyses were performed using the SPSS statistical program, version 16.0 (SPSS, Chicago, IL, USA). The data are shown as mean ± SD or percentage. Parameters of AUC and other relative clinic data were compared over groups of HbA1c using one-way ANOVA, followed by Bonferroni's test. Relative contributions of FG and PG were compared using a paired Student's *t* test. *P* < 0.05 was considered significant.

## 3. Results

The clinical characteristics and demographics of subjects with prediabetes and diabetes according to the quintiles of HbA1c are shown in Table 1. A total of 305 patients (208 men and 97 women) with newly diagnosed T2D or IGT/IFG were recruited. Their mean age was 51.1 ± 11.4 years, mean body mass index (BMI) was 25.3 ± 3.5 kg/m^2^, and mean HbA1c was 9.3 ± 1.9% (range 4.9–14.6%).

**Figure 1:**
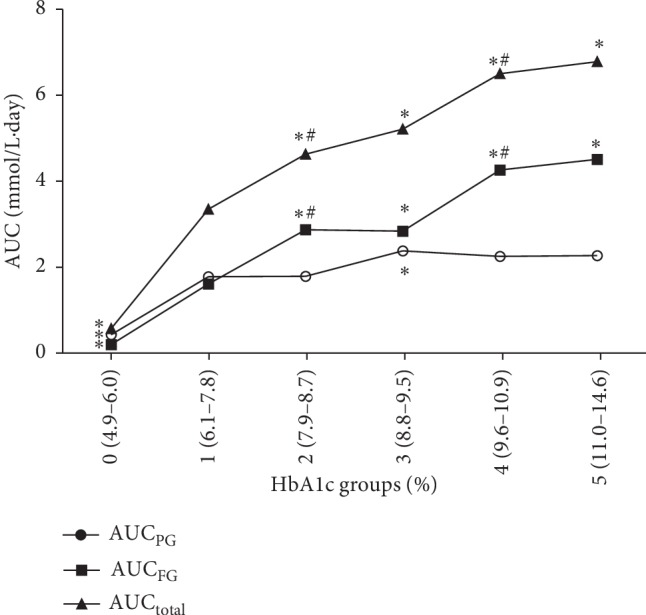
Area under the glucose curve derived from continuous glucose monitoring over groups of HbA1c. ^*∗*^Significant differences from group 1. ^#^Significant differences from the lower one group. Area under the glucose curve (AUC)_FG_ = AUC_total_ − AUC_PG_.

All the AUC results calculated from CGM are indicated in Table 1 and Figure 1. The level of FG, the overall hyperglycemia (AUC_total_), and fasting glucose increments (AUC_FG_) were increased from the lowest to the highest groups of HbA1c, especially in the higher groups. However, AUC_PG_, which reflects postprandial glucose increments, almost remained stable over the higher groups. In the lowest group, IGT/IFG group, AUC_PG_ and AUC_FG_ were both smallest. In lower group 1, AUC_PG_ was slightly above AUC_FG._ In quintiles 2 and 3, AUC_FG_ was higher than AUC_PG_ with the difference of 1.0 and 0.5 mmol/L·day, respectively. In the higher quintiles 4 and 5, the difference between AUC_FG_ and AUC_PG_ increased to 2.0 and 2.2 mmol/L·day, respectively.

**Figure 2:**
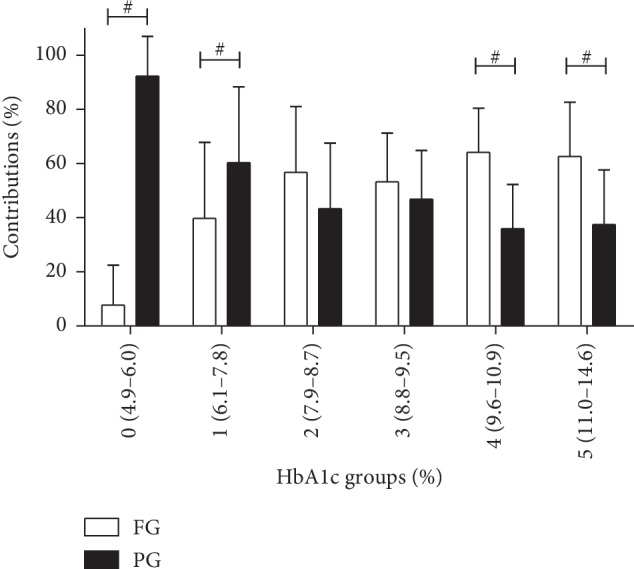
The relative contributions of fasting and postprandial glucose to overall glucose increment (%) over groups of HbA1c. ^#^Significant differences were observed between fasting and postprandial glucose (paired *t*-test). Area under the glucose curve (AUC)_FG_ = AUC_total_ − AUC_PG_.

The relative contribution of FG and PG is shown in Figure 2. PG contributed to hyperglycemia more than FG in the lowest HbA1c groups (range: 4.9–6.0%, mean: 5.5 ± 0.4% and range: 6.1–7.8%, mean: 7.3 ± 0.5%). FG and PG equally contributed to hyperglycemia in groups 2 and 3 (range: 7.9–8.7%, mean: 8.3 ± 0.3% and range: 8.8–9.5%, mean: 9.1 ± 0.2%). In the highest HbA1c quintiles 4 and 5 (range: 9.6–10.9%, mean: 10.1 ± 0.4% and range: 11.0–14.6%, mean: 12.1 ± 0.9%), fasting hyperglycemia began to play a major role in the contribution to hyperglycemia.

**Figure 3:**
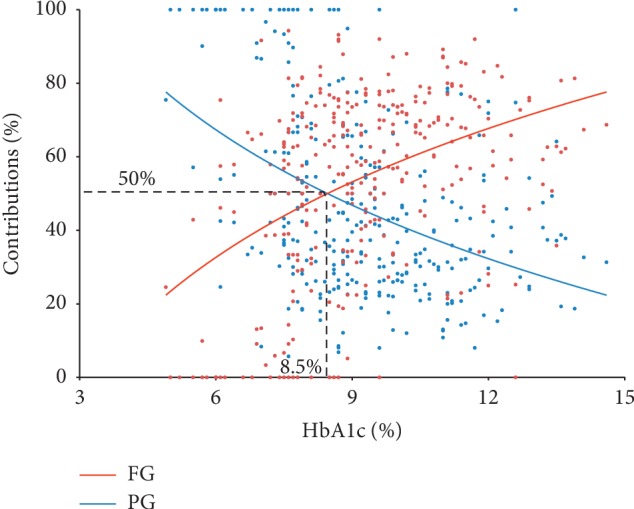
Correlation of the contribution of FG and PG and HbA1c.

As shown in Figure 3, the relative contribution of FG had obviously positive correlation with HbA1c. On the contrary, the relative contribution of PG was decreasing with increase in HbA1c. The regression analysis between the contribution of FG or PG and HbA1c showed that the two regression curves jointed at the point of HbA1c = 8.5%, suggesting that FG contributes more than PG from HbA1c > 8.5% and FG contributes less than PG when HbA1c < 8.5%.

## 4. Discussion

The present study indicates that in newly diagnosed, drug-naïve T2D or IGT/IFG, FG had dominant contribution to poorly controlled patients with HbA1c > 8.5%. Also, PG contributed more when HbA1c < 8.5%.

AUC_FG_ significantly increased from group 0 to 5, especially in the upper groups, whereas AUC_PG_ almost remained stable. It suggested that fasting hyperglycemia in the patients with higher HbA1c was the rising tide that would lift the postprandial hyperglycemia boat. When the fasting hyperglycemia decreased, the PG levels would drop down with the tide. Therefore, to eradicate hyperglycemia in poorly controlled patients with type 2 diabetes, the main principle of pharmacological intervention should be to choose therapeutic agents with an action primarily on basal glucose excursions. Although a recent review showed that PG strongly correlates with HbA1c or contributes significantly to overall glycemic control [17], FG should not be ignored when it comes to the management of patients with T2D. FG was also found to be more important than PG in diagnosed T2D treated by antihyperglycemic drugs with HbA1c ≥ 9.3% [6].

PG excursions in combination with FG play an important role in the contribution to overall hyperglycemia. Postprandial glucose was a strong predictor of cardiovascular events and all-cause mortality in a long-term follow-up [18]. Here, we found that the contribution of PG to overall hyperglycemia was higher than that of FG, when HbA1c ranged from 4.9% to 6.0% (mean: 5.5 ± 0.4%) in IGT/IFG patients and ranged from 6.1% to 7.8% (mean: 7.3 ± 0.5%) in patients with diabetes. Previous studies reported that mild hyperglycemia with HbA1c < 7.3% or 7% or between 6.5 and 6.9% inclusive was mainly attributed to the elevation of PG [6, 19, 20]. All of these published studies suggested that the therapeutic measures of patients with mild hyperglycemia should be aimed at reducing postmeal glucose.

Kang et al. [19] performed CGMS in 59 newly diagnosed, drug-naive patients with T2D patients and found that the contribution of PG was 57.78% which was significantly higher than FG when HbA1c ≤ 7%, whereas the contribution of FG was 79.58% significantly higher than PG when HbA1c > 9%, and the contribution of FG and PG was equal with HbA1c between 7 and 9%. However, the study sample was small. Here, in a larger population, we found similar association between the contribution of FG and PG and HbA1c. More importantly, we showed that FG contributes more than PG from HbA1c > 8.5% and FG contributes less than PG when HbA1c < 8.5%, providing a cut point for reference in clinical treatment.

In conclusion, our results indicate that in drug-naïve, Chinese patients with T2D or IGT/IFG, from mild to severe hyperglycemia, the predominant contribution to HbA1c changed from PG to FG with the changing point at HbA1c = 8.5%. This finding may aid doctors in formulating effective therapeutic plans according to the level of HbA1c.

## Figures and Tables

**Table 1:** Characteristics of all prediabetes and T2D patients over groups of HbA1c.

HbA1c Groups	IGT/IFG	Quintiles of glycated haemoglobin in T2D patients					Total
	0	1	2	3	4	5	
Number	10	61	55	55	62	62	305
Age (yrs)	41.1 ± 19.4	53.2 ± 10.9^*∗*^	53.3 ± 12.3	52.0 ± 9.3	50.3 ± 10.6	48.8 ± 10.8	51.1 ± 11.4
Sex (M/F)	5/5	42/19	40/15	36/19	40/22	45/17	208/97
Body mass index (kg/m^2^)	28.4 ± 8.3	25.7 ± 3.1	25.7 ± 3.2	25.9 ± 3.5	25.2 ± 2.7	23.9 ± 3.5	25.3 ± 3.5
Haemoglobin (g/L)	137.3 ± 13.9	142.9 ± 16.9	144.6 ± 14.6	144.7 ± 18.4	144.7 ± 13.2	141.1 ± 12.2	143.4 ± 15.1
Fasting glucose (mmol/L)	5.6 ± 0.9	7.8 ± 1.8^*∗*^	9.2 ± 2.0^*∗*^	9.2 ± 1.5	10.7 ± 2.0^*∗*^	11.1 ± 2.5	9.5 ± 2.4
AUC_total_ (mmol/L·day)	0.57 ± 0.45	3.35 ± 1.84^*∗*^	4.63 ± 2.05^*∗*^	5.21 ± 1.75	6.50 ± 2.29^*∗*^	6.78 ± 2.69	5.16 ± 2.60
AUC_FG_ (mmol/L·day)	0.20 ± 0.25	1.61 ± 1.49	2.87 ± 1.78^*∗*^	2.84 ± 1.44	4.26 ± 1.90^*∗*^	4.51 ± 2.38	3.14 ± 2.16
AUC_PG_ (mmol/L·day)	0.43 ± 0.43	1.78 ± 1.12^*∗*^	1.79 ± 0.96	2.38 ± 1.13	2.25 ± 1.12	2.27 ± 1.12	2.04 ± 1.14
FG contribution (%)	7.73 ± 14.69	39.71 ± 28.06^*∗*^	56.72 ± 24.27^*∗*^	53.22 ± 17.96	64.08 ± 16.32	62.54 ± 20.15	53.76 ± 24.64
PG contribution (%)	92.27 ± 14.69	60.29 ± 28.06^*∗*^	43.28 ± 24.27^*∗*^	46.78 ± 17.96	35.92 ± 16.32	37.47 ± 20.15	46.24 ± 24.64
Mean glycated haemoglobin (%)	5.5 ± 0.4	7.3 ± 0.5^*∗*^	8.3 ± 0.3^*∗*^	9.1 ± 0.2^*∗*^	10.1 ± 0.4^*∗*^	12.1 ± 0.9^*∗*^	9.3 ± 1.9
Range of glycated haemoglobin (%)	4.9–6.0	6.1–7.8	7.9–8.7	8.8–9.5	9.6–10.9	11.0–14.6	4.9–14.6

Data are mean ± SD. ^*∗*^Significant differences from the lower quintile.

## Data Availability

The data used to support the findings of this study are available from the corresponding author upon request.
